# Revisional bariatric surgery following sleeve gastrectomy: a meta-analysis comparing Roux-en-Y gastric bypass and one anastomosis gastric bypass

**DOI:** 10.1308/rcsann.2024.0054

**Published:** 2024-07-31

**Authors:** G Santoro, J Alfred, A Rehman, N Sheriff, H Naing, A Tandon

**Affiliations:** ^1^ Liverpool University Hospitals NHS Foundation Trust, UK; ^2^ Mersey and West Lancashire Teaching Hospitals NHS Trust, UK; ^3^ Warrington and Halton Hospitals Teaching NHS Foundation Trust, UK

**Keywords:** Revisional surgery, Sleeve gastrectomy, One anastomosis gastric bypass, Roux-en-Y gastric bypass

## Abstract

**Introduction:**

The number of bariatric operations is increasing each year. Sleeve gastrectomy is the most popular procedure; however, it often requires revision surgery because of insufficient weight loss, weight regain or gastro-oesophageal reflux disease (GORD). The most popular revisional procedures are Roux-en-Y gastric bypass (RYGB) and one anastomosis gastric bypass (OAGB). The primary outcome of this meta-analysis was weight loss after revisional surgery following laparoscopic sleeve gastrectomy and the secondary outcomes were gastro-oesophageal reflux, BMI difference, operative time, bleeding and anastomotic leak.

**Methods:**

A systematic electronic search was undertaken using PubMed, MEDLINE, Ovid, Cochrane Library and Google Scholar following PRISMA guidelines. The initial search identified 2,546 articles. After screening, seven papers met the inclusion criteria: six retrospective studies and one randomised controlled trial.

**Results:**

In total, 802 patients met the inclusion criteria: 390 had an OAGB and a further 412 had an RYBG. All patients previously had a sleeve gastrectomy for weight loss. The length of follow-up was 12 months for our primary outcome. We found no statistically significant difference in excess weight loss (%EWL) between OAGB and RYGB (*p* = 0.11). The incidence of postoperative reflux was statistically significantly higher in the OAGB group (16% vs 10.1%, *p* < 0.003). Operative time was statistically significantly lower in the OAGB group (*p* = 0.04).

**Conclusions:**

This meta-analysis showed no statistically significant difference between the two revision bariatric surgery procedures for %EWL. RYGB was superior to OAGB in reducing the incidence of symptomatic GORD, whereas OAGB had a significant shorter operative time.

## Introduction

The number of bariatric operations is rising each year, and this includes both primary and revisional surgeries. There is increasing acceptance of obesity as a chronic disease that cannot often be cured with a single intervention.^1^ However, in severe obesity bariatric surgery remains the best treatment modality, with beneficial effects on long-term weight loss, improved cardiovascular risk factors and reduced overall mortality.^2^ Laparoscopic sleeve gastrectomy (LSG) is the most common bariatric surgery worldwide, accounting for 50.2% of bariatric procedures as reported by the International Federation for the Surgery of Obesity and Metabolic Disorders in 2021.^3^

Recently, long-term follow-up studies have found a high rate of conversion to alternative bariatric procedures because of symptomatic gastro-oesophageal reflux disease (GORD), weight regain, insufficient weight loss or complications after sleeve gastrectomy.^4^ Several conversion procedures can be performed with various benefits; the two used most frequently are Roux-en-Y gastric bypass (RYGB)^5^ and one anastomosis gastric bypass (OAGB).^6^

Although these procedures have been shown to be effective as primary surgeries for obesity, there is a lack of high-quality studies and long-term follow-up evaluating their efficacy as revisional surgery.

## Methods

### Eligibility criteria

A systematic electronic search was undertaken using PubMed, MEDLINE, Ovid, Cochrane Library and Google Scholar following Preferred Reporting Items for Systematic Reviews and Meta-Analyses (PRISMA) guidance.^7^ Search terms comprised the keywords ‘revisional surgery’, ‘sleeve gastrectomy’, ‘Roux-en-Y gastric bypass’ and ‘one anastomosis gastric bypass’. Database searches were performed using a predefined, peer-reviewed search for maximum sensitivity. All search terms were combined with Boolean operators and searched as both keywords and Medical Subject Headings. The results were reviewed by two independent researchers (GS and JA). The last search date was 12 March 2024.

### Inclusion and exclusions criteria

All randomised and non-randomised studies that reported the following criteria were included: a patient who had revisional bariatric surgery following failure of LSG and all studies comparing OAGB vs RYGB as revisional surgery.

Studies were excluded if they were case reports, small case series of fewer than ten patients, conference abstracts or letters to editors. Non-human and non-English studies were also excluded.

### Outcome measures

The primary outcome was weight loss after revisional surgery. This was expressed as %EWL in patients undergoing RYGB or OAGB as a revisional bariatric surgery following unsatisfactory weight loss or weight regain after LSG. This was defined as insufficient weight loss with EWL <50% or weight regain >15% following LSG.

Secondary outcomes were de novo incidence of gastroesophageal reflux, pre- and post-revisional body mass index (BMI), operative time, intraoperative complications like bleeding and anastomotic leak, and resolution of comorbidities.

### Search strategy

**Figure 1:**
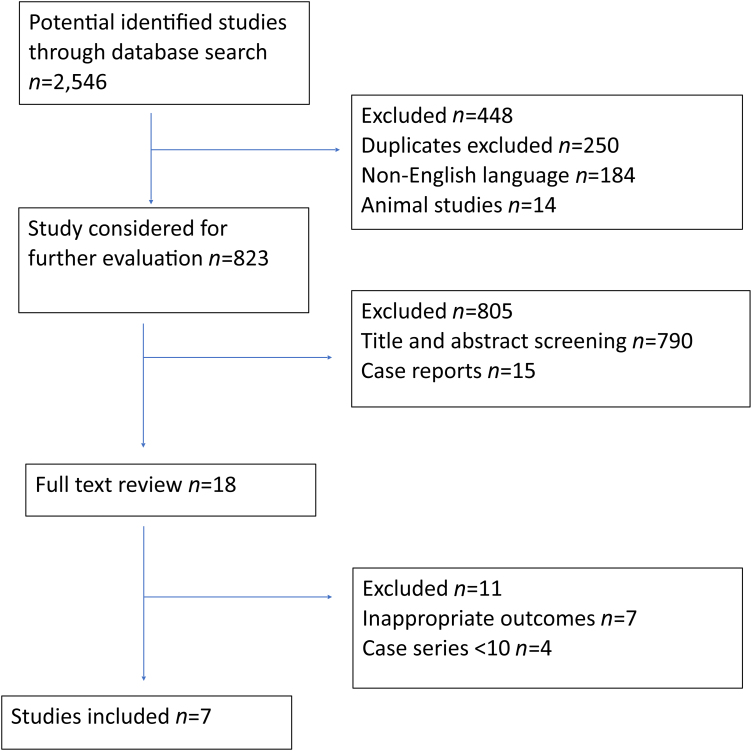
PRISMA diagram

Two authors independently reviewed all the abstracts identified using the search strategy and excluded studies that did not meet the inclusion criteria. Where it was not possible to screen the article from the abstract alone, the full text was reviewed. During the selection process, any differences of opinion between the two authors were resolved by consensus with the senior author (AT). Additional papers were detected by screening the references of relevant papers. Relevant titles were included in the search results, and these papers were read in full. Papers for which the full article texts were not available in English were excluded. A summary of the included papers is displayed in the PRISMA diagram (Figure 1).

### Data extraction

Data were extracted on study characteristics including the study year, first author, study design, number of patients, mean time to follow-up and patient demographics (age, sex). Primary and secondary outcomes in the two groups were collected by the primary author (GS) using a data extraction form and were reviewed by the senior author (AT).

### Statistical analysis and quality assessment

**Figure 2:**
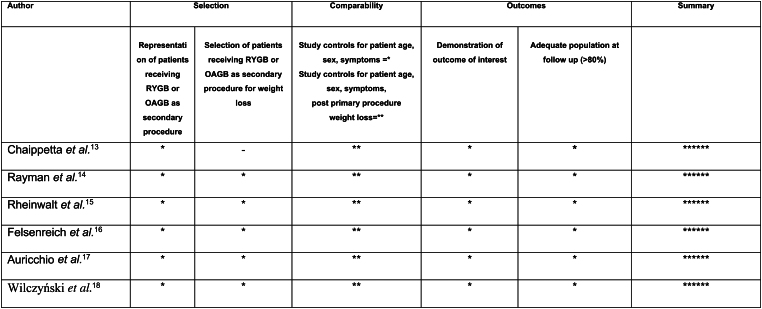
Modified Newcastle–Ottawa Scale judgement

Statistical analyses were undertaken using RevMan version 5.3 (The Cochrane Collaboration, The Nordic Cochrane Centre, Copenhagen, Denmark). Data were pooled, and risk ratios (RRs) and standardised mean differences (SMDs) were calculated with their 95% confidence intervals (CI). A random effect model was used for meta-analysis to account for heterogeneity among the studies.^8^ Heterogeneity was tested and reported using the *I*^2^ value. All studies reported the published outcomes. For non-randomised controlled studies, risk of bias on selection, comparability and outcome assessment was performed using a modified version of the Newcastle–Ottawa Scale (Figure 2).^9^

## Results

### Results of the literature search

The initial literature search identified 2,546 articles. Following exclusion of duplicates, animal studies and non-English articles, 823 studies were selected for evaluation. Of these, 790 articles were excluded after title and abstract review, with a further 15 exclusions of case reports. Of the remaining 18 studies, after full-text review, 11 were excluded because they did not report outcomes of interest or had a small population. Of the final seven studies selected (Table 1), six were retrospectives and one was a randomised controlled trial (RCT).^10–16^

**Table 1:** Study characteristics

Author	Year	Country	Journal	Design	Follow-up (months)
Chiappetta *et al*^10^	2018	Germany/Italy	*Obesity Surgery*	Retrospective	12
Rayman *et al*^11^	2021	Israel	*Obesity Surgery*	Retrospective	32
Rheinwalt *et al*^12^	2022	Germany	*World Journal of Surgery*	Retrospective	24
Felsenreich *et al*^13^	2022	Austria	*Obesity Surgery*	Retrospective	60
Auricchio *et al*^14^	2022	Italy	*Surgeries*	Retrospective	12
Wilczyński *et al*.^15^	2022	Poland	*Journal of Gastrointestinal Surgery*	Retrospective	60
Hany *et al*^16^	2022	Egypt	*Obesity Surgery*	Randomised controlled trial	24

### Study characteristics

A total of 802 patients met the inclusion criteria: 390 OAGB and 412 RYGB. All patients previously had LSG for weight loss or weight regain. The length of follow-up ranged from 12 to 60 months. The baseline characteristics of the included studies and population demographics are given in Table 2.

**Table 2:** Baseline characteristics

Author	Study size	Age (mean)	Sex (F:M)	Primary procedure	Secondary procedure (OAGB– RYGB)
Chaippetta *et al*^10^	55	46	32:13	LSG	34:21
Rayman *et al*^11^	263	44	191:72	LSG	144:119
Rheinwalt *et al*^12^	123	44	89:34	LSG	55:68
Felsenreich *et al*^13^	58	–	–	LSG	13:45
Auricchio *et al*^14^	63	39	56:7	LSG	17:46
Wilczyński *et al*^15^	77	45	34:13	LSG	47:33
Hany *et al*^16^	176	42	138:22	LSG	80:80

LSG = Laparoscopic sleeve gastrectomy; OAGB = one anastomosis gastric bypass; RYGB = Roux-en-Y gastric bypass.

### Difference in BMI pre- and post-revisional surgery

**Figure 3:**
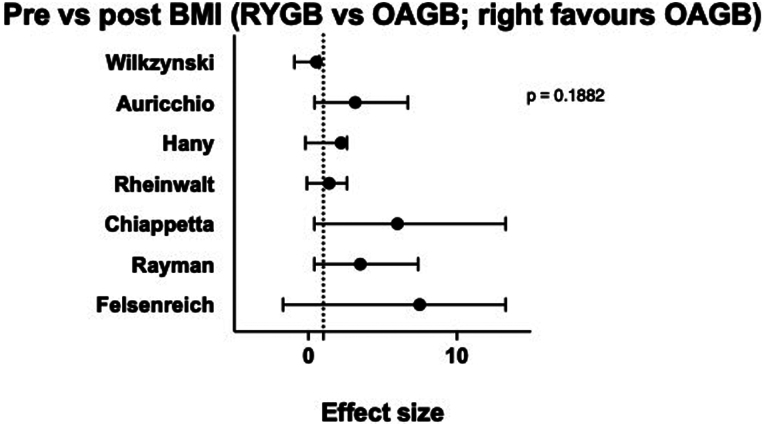
Difference in BMI pre- and post revisional surgery

Pre- and post-revisional surgery BMI were reported in all seven studies (Table 3).^10–16^ A total of 802 patients were included (390 OAGB and 412 RYGB), with no statistically significant difference between the two groups (*p* = 0.1882) (Figure 3).

**Table 3:** Pre- and post-revisional surgery BMI loss

Author	Pre-revisional BMI (kg/m^2^)		Post-revisional BMI (kg/m^2^)	
	OAGB	RYGB	OAGB	RYGB
Chiappetta *et al*^10^	45.7 ± 8	36.6 ± 6.9	36.6 ± 6.3	33.5 ± 5.6
Rayman *et al*^11^	41.6 ± 5.7	39.6 ± 5.0	31.8 ± 5.3	33.3 ± 5.0
Rheinwalt *et al*^12^	45.5	39.3	31	35
Felsenreich *et al*^13^	45.0 ± 7.3	38.6 ± 8.6	30.3 ± 8.5	31.4 ± 8.1
Hany *et al*^16^	45.1 ± 8.3	44.9 ± 6.6	27.8 ± 2.2	27.4 ± 3.1

MI = body mass index; OAGB = one anastomosis gastric bypass; RYGB = Roux-en-Y gastric bypass.

### Excess weight loss

**Figure 4:**
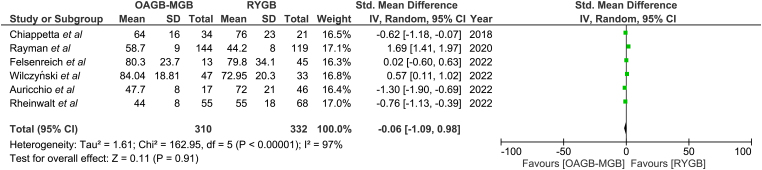
Excess weight loss

Excess weight loss was reported in six papers for a total of 642 patients.^10–15^ There were 310 patients in the OAGB group and 332 patients in the RYGB group (Figure 4). At 12-month follow-up, there was no statistically significant difference between OAGB and RYGB (SMD 0.06, 95% CI −1.09, 0.98; *p* = 0.11). Heterogeneity among the studies was high at 97%.

### Anastomotic leak

**Figure 5:**
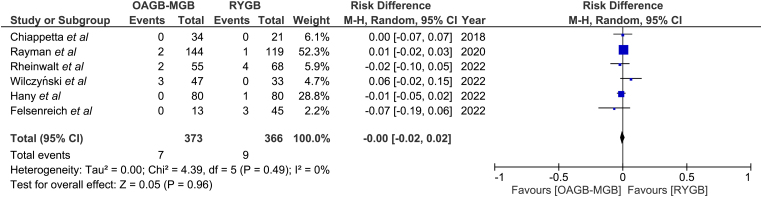
Anastomotic leak

Anastomotic leak was reported in six studies for a total of 739 patients (Figure 5).^10–13,15,16^ The OAGB group reported 7 of 373 (1.8%) leaks, whereas the RYGB group had 9 of 366 (2.4%) leaks. There was no statistically significant difference in anastomotic leak rate between the groups (risk difference, RD 0.00, 95% CI −0.02, 0.02; *p* = 0.96). There was no heterogeneity among the studies (*I*^2 ^= 0%).

### Postoperative reflux

**Figure 6:**
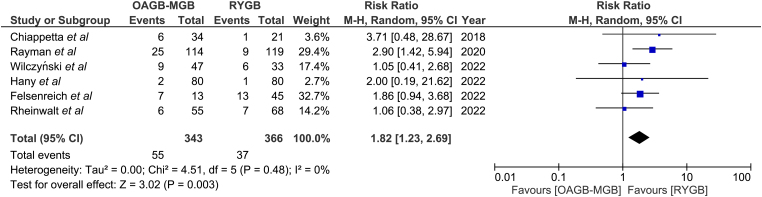
Postoperative reflux

Postoperative reflux was reported in six papers (Figure 6).^10–13,15,16^ The incidence of de novo postoperative reflux was higher in the OAGB group than in the RYGB group [55 of 343 (16%) vs 37 of 366 (10.1%)]. This difference was statistically significant (*p* < 0.003) in favour of RYGB (RR 1.82, 95% CI 1.23, 2.69). There was no heterogeneity among the studies (*I*^2 ^= 0%).

### Postoperative bleeding

**Figure 7:**
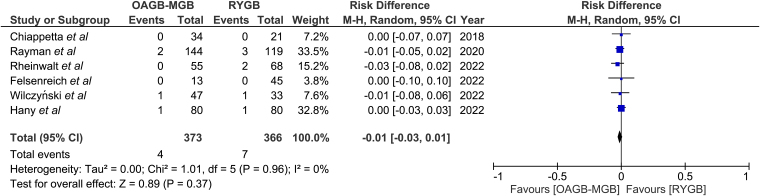
Postoperative bleeding

Postoperative bleeding was reported in six studies, for a total of 739 patients (Figure 7).^10–13,15,16^ Postoperative bleeding requiring intervention was similar in the RYGB and OAGB groups (1% vs 1.9%). This difference was not statistically significant (RD −0.01, 95% CI −0.03, 0.01; *p* = 0.37). There was no heterogeneity among the studies (*I*^2^ = 0%).

### Operative time

**Figure 8:**
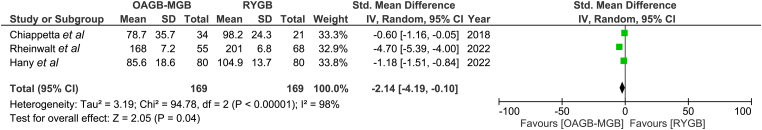
Operative time

Operative time was reported in three studies, for a total of 338 patients (Figure 8).^10,12,16^ The remaining three studies did not adequately report operative times. Operative time was significantly lower in OAGB compared with RYGB (SMD −2.14, 95% CI −4.19, −0.10; *p* = 0.04). There was a high level of heterogeneity among the studies (*I*^2^ = 98%).

### Comorbidity resolution

Resolution of comorbidities was reported in six papers.^12,13,15,16^ The three comorbidities documented were diabetes, hypertension and obstructive sleep apnoea. The OAGB group had a greater resolution of diabetes (79.5% vs 61.2%, *p* = 0.1708), obstructive sleep apnoea (75.0% vs 64.3%, *p* = 0.2563) and hypertension (67.3% vs 48.5%, *p* = 0.3199) compared with the RYGB group. None of the differences were statistically significant. The outcome is reported in Tables 4 and 5.

**Table 4:** Pre- and post-revision GORD

Author	Pre-revision GORD (%)		Post-revision GORD (%)	
	OAGB	RYGB	OAGB	RYGB
Chiappetta *et al*^10^	–	–	11.8	4.8
Rayman *et al*^11^	19.5	34.5	17.4	7.6
Rheinwalt *et al*^12^	81.8	92.6	10.9	10.2
Felsenreich *et al*^13^	–	–	53.8	28.9
Auricchio *et al*^14^	23	45	–	–
Wilczyński *et al*^15^	15	30	19	18
Hany *et al*^16^	41.3	40	2.5	1.5

GORD = gastro-oesophageal reflux disease; OAGB = one anastomosis gastric bypass; RYGB = Roux-en-Y gastric bypass.

**Table 5:** Comorbidities resolution in percentage

Author	Diabetes (%)		Hypertension (%)		Obstructive sleep apnoea (%)	
	OAGB	RYGB	OAGB	RYGB	OAGB	RYGB
Chiappetta *et al*^10^	100	60	66.7	0	0	80
Rayman *et al*^11^	65	45	46.6	12.5	84	84
Rheinwalt *et al*^12^	92	100	92	89	82	65
Felsenreich *et al*^13^	80	90	88	65	80	80
Wilczyński *et al*^15^	91.7	33	27.3	30	60	100
Hany *et al* ^16^	75	71.4	63	75	–	–
Total events	66/83 (79.5%)	41/67 (61.2%)	68/101 (67.3%)	50/103 (48.5%)	27/36 (75.0%)	27/42 (64.3%)
*p-*value	0.1708		0.3199		0.2563	

OAGB = one anastomosis gastric bypass; RYGB = Roux-en-Y gastric bypass.

## Discussion

Revisional surgery is a growing subset of bariatric surgical procedures. As the numbers of bariatric procedures increase so does the need for revisional surgery.^17^ Currently, revisional surgery represents 7–15% of the total number of bariatric operations with 9–11% being performed after sleeve gastrectomy.^18–20^

Despite this increasing popularity, there remains a significant lack of published long-term follow-up in this patient cohort; what is more, the potential risk of complications is relatively high when compared with primary bariatric procedures.^17,21^

One of the most popular bariatric procedures worldwide is LSG.^21^ However, this procedure is not without its complications and failure rate. The most common issues with LSG are related to weight regain and insufficient weight loss, as well as worsening of GORD.^22^ DuPree *et al* in their review of 4,832 patients who underwent an LSG demonstrated that this procedure did not reliably relieve or improve reflux symptoms.^23^ In fact, reflux was induced in some patients who were previously asymptomatic. The authors went on to suggest that preoperative reflux symptoms might be a contraindication to a sleeve procedure. Similarly, Vitiello *et al* in a 5-year follow-up of 66 patients who underwent LSG found a new onset of reflux symptoms in 66.7% of cases.^24^

Moreover, the 5-year review by Sebastianelli *et al* found that there was a prevalence of GORD, erosive gastritis and increased use of proton pump inhibitors following LSG. They also demonstrated the prevalence of Barrett’s oesophagus in 18.8% of patients.^25^

Nevertheless, it has been noted in the literature that patients who have had insufficient weight loss from previous bariatric surgery can go on to have good outcomes following revisional surgery.^26,27^ A 10-year review by Lazzati *et al* showed a 12.2% revision rate following LSG, with the most common revision procedure being RYGB (75.2%), followed by re-sleeve (18.7%). Notably, OAGB is gaining popularity both as primary and revisional surgery.^28,29^

In a recent RCT, OAGB has been shown to be superior at 5-year follow-up for excess BMI lost (EBMI) and comorbidity resolution when compared with LSG and RYGB as primary procedures.^30^

In a review of 56 patients who had OAGB following a gastric sleeve, Jamal *et al* observed that their patients achieved 24% total weight loss (TWL) at 19 months.^31^

Looking at the studies used in this meta-analysis, Chiappetta *et al* found in their retrospective study that %TWL was significantly greater in the revisional OAGB group than in the revisional RYGB group at 1-year follow-up.^10^ By contrast, a retrospective study by Rheinwalt *et al* in 2022 found more comparable findings for %TWL, comorbidity resolution and GORD between OAGB and RYGB as conversional procedures following failure of LSG.^12^ Although a significantly shorter operative time was reported for OAGB, similar results were observed by Felsenreich *et al* in 2021 in their retrospective study of 58 patients, which concluded that both OAGB and RYGB following LSG were equal in terms of additional weight loss and remission of comorbidities.^13^ However, in this study, RYGB was superior in remission of clinical GORD compared with OAGB.

Conversely, Auricchio *et al* in a retrospective study comparing RYGB and OAGB after LSG found that RYGB was superior to OAGB in terms of weight loss.^14^

In addition, results from a single-blinded RCT by Hany *et al*, including 176 patients, comparing the two procedures (OAGB and RYGB) post LSG showed no statistically significant difference in %EBMIL at 1 and 2 years of follow-up.^16^ Outcomes were similar for comorbidity resolutions and complications rate.

The pooled data from the included studies in our meta-analysis have shown that OAGB and RYGB as revisional procedures are comparable for EWL% and EBMI loss at 1-year follow-up. It was unsurprising that RYGB had a significantly lower incidence of de novo GORD compared with OAGB. As shown in Table 4, the incidence of pre-revision GORD was higher, in most studies, for the RYGB group, thus introducing selection bias.

OAGB operative time was significantly shorter, with a significantly lower incidence of postoperative bleeding when compared with RYGB.

Lastly, at 3 years of follow-up, we observed no significant difference in the resolution of comorbidities between OAGB and RYGB, which is in keeping with previous studies.^32,33^

This analysis supports the use of OAGB as a valid alternative to RYGB in improving long-term outcomes in the comorbid population of bariatric patients.^34^

### Study limitations

There are many limitations to our meta-analysis. First, when reporting the primary outcome, we could only include retrospective studies, leading to potential selection bias. This also introduces the potential for selection bias both due to the lack of RCTs reporting the outcome of interest, and also because of less-strict selection criteria in non-randomised studies. The latter, however, makes them more representative of clinical practice.^35^

Second, the authors also intentionally limited the primary outcome follow-up at 12 months to reduce transfer bias, thus it was not possible to evaluate long-term outcomes. Third, there was a high level of heterogeneity between studies; this was due to a varied population and a wide range of indications for revisional surgery. Finally, we must also consider the risk for ascertainment bias related to the occurrence reporting of GORD which, although confirmed endoscopically in three studies, was solely based on symptoms assessment in the other two.

A longer follow-up comparing the two revisional procedures as well as further high-quality prospective studies are needed to improve the evidence base around this topic and ultimately assist clinicians in supporting their patients to make informed decisions about their surgical options.

## Conclusions

This meta-analysis has shown comparable results for excess weight loss at 1-year follow-up between OAGB and RYGB when performed as revisional procedures following LSG. This study also observed a significantly lower incidence of postoperative GORD in the RYGB group, albeit with a significantly longer operative time compared with OAGB.

At 3-year follow-up, OAGB was superior to RYGB in resolution of diabetes, hypertension and obstructive sleep apnoea; however, the difference were not statistically significant.
